# Landscape of Immune Microenvironment in Epithelial Ovarian Cancer and Establishing Risk Model by Machine Learning

**DOI:** 10.1155/2021/5523749

**Published:** 2021-08-26

**Authors:** Shi-yi Liu, Rong-hui Zhu, Zi-tao Wang, Wei Tan, Li Zhang, Yan-qing Wang, Fang-fang Dai, Meng-qin Yuan, Ya-jing Zheng, Dong-yong Yang, Fei-yan Wang, Shu Xian, Juan He, Yu-wei Zhang, Ma-li Wu, Zhi-min Deng, Min Hu, Yan-xiang Cheng, Ye-qiang Liu

**Affiliations:** ^1^Department of Obstetrics and Gynecology, Renmin Hospital of Wuhan University, Wuhan 430060, Hubei, China; ^2^Shanghai Skin Disease Clinical College of Anhui Medical University, Shanghai Skin Disease Hospital, Shanghai 200443, China; ^3^Department of Dermatopathology, Shanghai Skin Disease Hospital, Tongji University School of Medicine,, Shanghai 200071, Shanghai, China

## Abstract

**Background:**

Epithelial ovarian cancer (EOC) is an extremely lethal gynecological malignancy and has the potential to benefit from the immune checkpoint blockade (ICB) therapy, whose efficacy highly depends on the complex tumor microenvironment (TME).

**Method and Result:**

We comprehensively analyze the landscape of TME and its prognostic value through immune infiltration analysis, somatic mutation analysis, and survival analysis. The results showed that high infiltration of immune cells predicts favorable clinical outcomes in EOC. Then, the detailed TME landscape of the EOC had been investigated through “xCell” algorithm, Gene set variation analysis (GSVA), cytokines expression analysis, and correlation analysis. It is observed that EOC patients with high infiltrating immune cells have an antitumor phenotype and are highly correlated with immune checkpoints. We further found that dendritic cells (DCs) may play a dominant role in promoting the infiltration of immune cells into TME and forming an antitumor immune phenotype. Finally, we conducted machine-learning Lasso regression, support vector machines (SVMs), and random forest, identifying six DC-related prognostic genes (*CXCL9*, *VSIG4*, *ALOX5AP*, *TGFBI*, *UBD*, and *CXCL11*). And DC-related risk stratify model had been well established and validated.

**Conclusion:**

High infiltration of immune cells predicted a better outcome and an antitumor phenotype in EOC, and the DCs might play a dominant role in the initiation of antitumor immune cells. The well-established risk model can be used for prognostic prediction in EOC.

## 1. Introduction

Ovarian cancer is the second leading cause of cancer death in adult women, and the epithelial ovarian cancer (EOC) is the most common histological subtype characterized by complex adjacent anatomical structures, high-degree malignancy, and heterogeneity [1, 2]. The surgical excision and chemotherapy are effective for EOC patients, but a large fraction of EOC patients will subsequently relapse and develop to chemoresistance, which seriously shortens the patients' clinical survival [3].

Nowadays, the immune checkpoint blockades (ICBs) have received wide attention and have emerged as efficient agents for tumor therapy. It can specifically block the immune checkpoints and retrieve the antitumor immunity [4]. Nevertheless, due to the lack of comprehensive analysis on the tumor microenvironment (TME) and EOC, there is a limited application of ICBs in EOC therapy.

The TME not only plays a critical role in carcinogenesis but also has various regulatory functions in tumor growth and metastasis [5]. It has been proposed to be valuable in diagnosis and prognosis in a wide range of tumors, but the utility on EOC has not been investigated in detail [6]. The immune cells are a master component in TME, composed of CD8^+^ cytotoxic T lymphocytes (CTLs), B cells, plasma cells, macrophages, and dendritic cells (DCs) [7–9]. The class II major histocompatibility complex- (MHC-I-) restricted CTLs implicated as critical components in antitumor immunity, contributing to tumor killing [10]. And CD4^+^ T*-*helper (Th) cells are an important helper in the activation of CTLs. CTLs also can indirectly mediate tumor killing by the secretion of lymphokines such as gamma-interferon (IFN-*γ*), lymphotoxin [11]. In addition, DCs, a professional antigen presenting cells (APCs), are central regulators in the T-cell-mediated antitumor immunity [12]. Conversely, tumor-intrinsic immune checkpoints' ligand like programmed death ligand-1 (PD-L1) can inhibit the activation of CTLs via binding to its receptor PD-1, resulting in the tumor immune evasion [8, 13].

In this study, we intended to investigate the potential role and underlying mechanisms of TME in EOC. Immune infiltration analysis has indicated that the EOC patients with high-intensity infiltrating immune cells companied with better clinical outcomes, higher TMB, CSMD3, and MUC16 mutation. In addition, immune score was significantly related to antitumor immunity and the efficacy of ICBs in EOC. We emphasized that infiltrating dendritic cells plays a leading role in this antitumor immunity. Furthermore, we identified six DC-related prognostic genes for establishing the DC-related risk model using machine-learning Lasso regression, support vector machines (SVMs), and random forest. The well-established DC-related risk model in this study can accurately stratify patients into subgroups with different clinical survival.

## 2. Materials And Methods

### 2.1. Data

The high-throughput sequencing FPKM data (HTSeq-FPKM, *N* = 365) and corresponding clinicopathologic data of EOC were obtained from The Cancer Genome Atlas (TCGA, https://www.cancer.gov/). The HTSeq-FPKM data received a logarithmic conversion. In addition, Gene Expression Omnibus (GEO, http://www.ncbi.nlm.nih.gov/geo) databases GSE63885 (GPL570) [14], GSE32062 (GPL6480) [15], GSE105437 (GPL570) [16], GSE4122 (GPL201), and GSE23554 (GPL96) [17] were also included in our study, which were retrieved and downloaded by R programming using package “GEOquery”. GSE32062, GSE63885, and GSE23554 were measured in 260, 75, and 28 EOC patients, respectively; while the GSE105437 include 10 EOC tissues and five normal tissues; GSE4122 cohort was measured in 32 normal/benign and 32 malignant ovarian tissues.

### 2.2. Immune Infiltration Analysis

The “ESTIMATE” (Estimation of STromal and Immune cells in MAlignant Tumor tissues using Expression data) algorithm (R package “estimate”) was carried out to calculate the immune score, which refers to the infiltration level of total immune cells [18]. The populations of major types of infiltrating immune cells were evaluated through “xCell” (R package “xCell”) [19].

### 2.3. Somatic Mutation Analysis

The mutation MAF (minor allele frequency) file of EOC was downloaded through R package “TCGAbiolinks”. The total number of somatic mutations in each sample from TCGA was extracted through R package “maftools”. In TCGA, GRCh38 is the reference genome with a length of about 35 Mb, and the formula for calculating the tumor mutation burden (TMB) is as follows [20, 21]: TMB = Sn/35 (where Sn represents the total number of somatic mutations). The R package “maftools” was also used to plotting.

### 2.4. Gene Set Variation Analysis

All gene sets in this study for gene set variation analysis (GSVA) were obtained from the molecular signatures database (MSigDB, https://www.gsea-msigdb.org/gsea/msigdb, Supplementary Table 1). The GSVA score of each gene set was calculated using R package “GSVA” with ssGSEA (single-sample gene set enrichment analysis) method [22, 23].

### 2.5. Differential Expression Analysis

EOC patients were classified into high-DC and low-DC infiltration groups based on the median level of infiltrating DCs. R package “Limma” was conducted for differential expression analysis. Absolute values of log_2_ fold change (log FC) > 1 and adjusted *p* (adj.*p*) value < 0.05 were used as thresholds to identify differentially expressed genes (DEGs) [24].

### 2.6. Construction of Immune Molecular Risk Model

Univariable Cox survival analysis was performed to screen the potential prognostic genes (adj.*p* < 0.05). Feature selection was next applied by Lasso regularization (R packages “glmnet”), SVM (R packages “e1071”), and random forest (R package “randomForestSRC”, with variable relative importance > 0.4) [25]. Finally, a DC-related risk model was established by multivariable Cox analysis. The risk score was calculated as follows: risk score = *CXCL9 ∗* (−0.112) + *VSIG4 ∗* (0.173) + *ALOX5AP ∗* (0.105) + *TGFBI ∗* (0.077) + *UBD ∗* (−0.141) + *CXCL11 ∗* (−0.120).

### 2.7. Statistics

All statistics were performed in R program (version 4.0.0). Student's *t*-test or Wilcox test (according to the Shapiro test and Bartlett test) was applied to calculate the *p* value between two groups with continuous variables. Kaplan–Meier analysis was used for survival analysis, and the statistical significance was tested using the log-rank test. The correlation between two variables was assessed by the spearman correlation analysis. *p* < 0.05 was considered statistically significant.

## 3. Results

### 3.1. High Infiltration of Immune Cells Predicts Favorable Clinical Outcomes in Epithelial Ovarian Cancer

**Figure 1:**
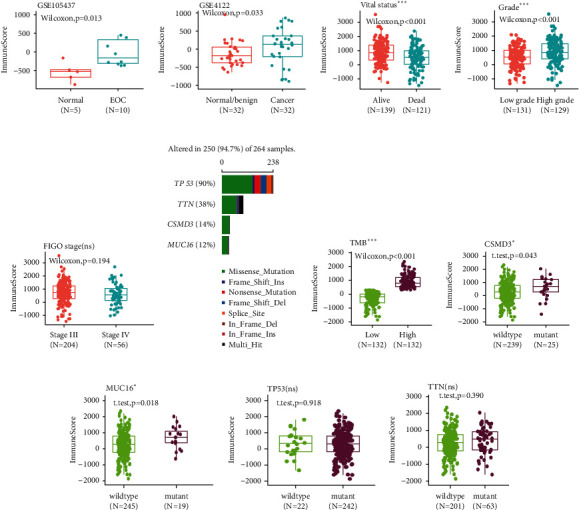
High infiltrating immune cells predict favorable clinical outcomes in epithelial ovarian cancer. (a) The distribution of immune score between EOC (*N* = 10) and normal tissues (*N* = 5) in GSE105437. Box plot shows that the immune score of EOC is significantly higher than normal tissues (Wilcoxon test, *p*=0.013). (b) In GSE4122 dataset, the immune score was higher in malignant ovarian tissues (Wilcoxon test, *p*=0.033). (c–e) Box plots of immune score in EOC patients from GSE32062, stratified by (c) vital status, (d) grade, and (e) FIGO stage. (f) Top four mutated genes in EOC from TCGA. (g–h) Box plots of immune score in EOC patients, stratified by TMB, CSMD3, MUC16, TP53, and TTN. EOC: epithelial ovarian cancer; TMB: tumor mutation burden.

To better understand the role of the infiltrating immune cells in EOC progression, the immune score based on the gene expression profiles was calculated using the “ESTIMATE” algorithm. As shown in results from GSE105437 and GSE4122, EOC patients had significantly higher immune score than normal tissues (Figure 1(a) and 1(b)). This result was consistent with our previous findings that immune cells have been increasingly infiltrated into tumor microenvironment [26]. In GSE32062, the alive patients had higher immune score than the dead (Figure 1(c), *p* < 0.001). In contrast, patients with high grade (Grades 3 and 4) demonstrated significantly higher immune score (Figure 1(d), *p* < 0.001), while the immune score in patients with FIGO stage III and stage IV did not differ significantly (Figure 1(e)).

Regarding the most common mutation like TP53 and BRCA1/2 in EOC [27], we plotted the distribution of the immune score across other frequently mutated gene TTN, CSMD3, and MUC16 in TCGA mutation data (Figure 1(f)). Patients with high immune score had higher TMB, CSMD3, and MUC16 mutation than in wild type (Figure 1(g)–1(i), *p* < 0.05). However, there were no significant correlations between immune score and mutations of TP53, TTN, BRCA1/2 (Figure 1(i)–1(k), Supplementary Figure 1).

**Figure 2:**
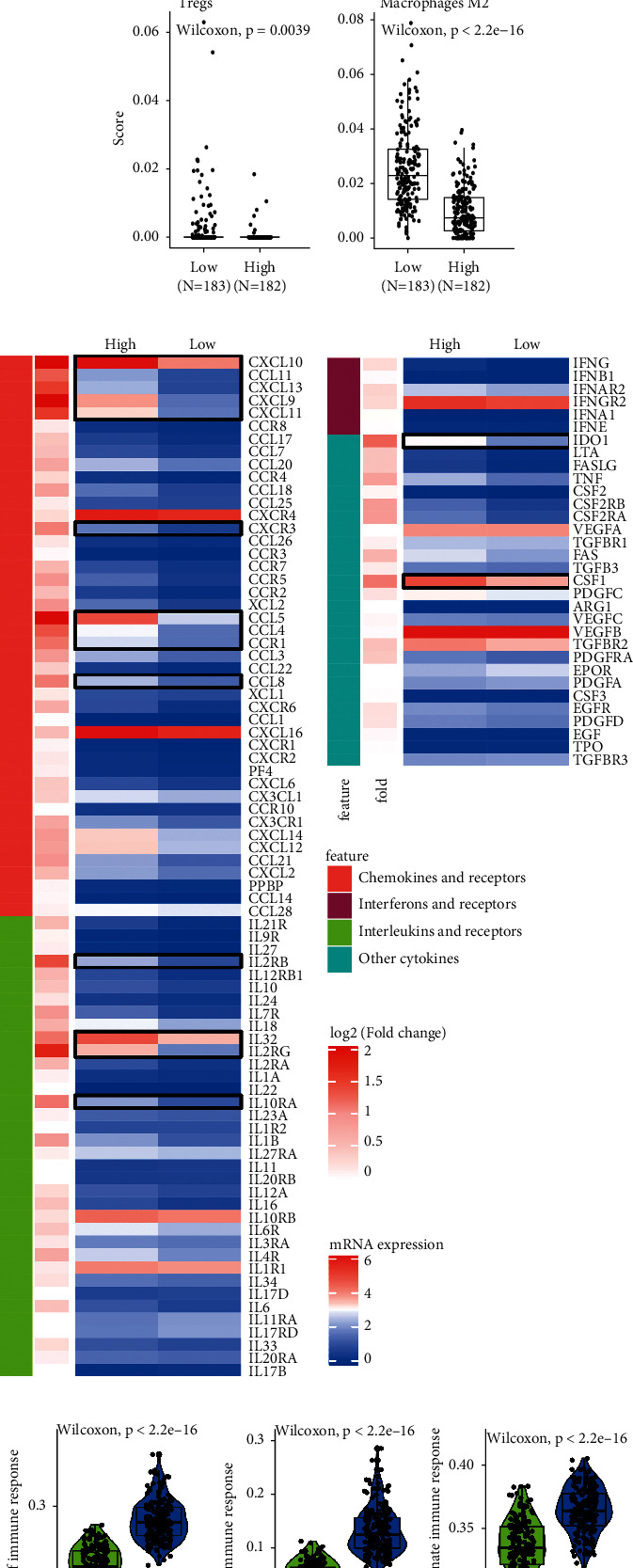
EOC patients with high infiltrating immune cells have an antitumor phenotype. (a–c) The high immune score was related to a better clinical survival in GSE32062 and GSE63885. Infiltration levels of (d) antitumor lymphocytes (CD8 + T cells, B cells, DCs, and macrophages M1) and (e) immunosuppressive cells (Tregs and macrophages M2) in high- and low-immune-score group. (f) Heatmap shows the expression of specific cytokines in high- and low-immune-score group. (g) Violin plots illustrate the enrichment scores of immune response biological processes evaluated by GSVA analysis between high- and low-immune-score groups. (h) Correlation between immune score and immune checkpoints, color represents the spearman's correlation coefficient. DC: dendritic cell; GSVA: gene set variation analysis.

To evaluate the potential prognostic utility of the infiltrating immune cells in EOC, the patients were initially classified into high and low groups according to the median immune score. In GSE32062 cohort, the median OS of the high and low groups were 69 and 50 months (Figure 2(a), *p*=0.01). And the immune score also was an indicator of longer progression-free survival (PFS) (Figure 2(b)). Moreover, GSE63885 also confirmed that the high immune score was correlated with better OS (Figure 2(c)). The prognostic accuracy of immune score to predict the OS in GSE32062 and GSE63885 were assessed via receiver operating characteristic (ROC) curve curves, as shown in Supplementary Figure 2, The area under the curve (AUC) value indicated that the immune score can be used to predict OS in EOC patients (Supplementary Figure 2).

### 3.2. EOC Patients with High Infiltrating Immune Cells Have an Antitumor Phenotype

The dynamic counterbalance between immunity and evasion in TME contributes to diverse immune phenotype, which is affected by lots of factors, including the infiltrating immune cells, cytokines, and immune checkpoints [28]. “xCell” algorithm was applied to quantify the different types of infiltrating immune cells. We observed high density of antitumor immune cells in high-immune-score group, including CD8^+^ T cells, B cells, DCs, and macrophages M1 (Figure 2(d), all *p* < 0.001). Conversely, immunosuppressive cells Tregs and macrophages M2 were more accumulated in the low group (Figure 2(e), all *p* < 0.01). Besides, 16 immunostimulatory-related cytokines were overexpressed in patients with high infiltrating immune cells, which include chemokines and receptors (*CXCL10*, *CCL11*, *CXCL13*, *CXCL9*, *CXCL11*, *CXCR3*, *CCL5*, *CCL4*, *CCR1*, and *CCL8*), interferons and receptors (IL2RB, IL32, IL2RG, and IL10RA), and other cytokines (IDO1 and CSF1) (Figure 2(f), |log FC| > 1, *p* < 0.05). Moreover, the GSVA scores of innate immunity and adaptive immunity were significantly correlated with infiltrating immune cells (Figure 2(g), all *p* < 0.001). We defined that EOC patients with high infiltrating immune cells trend to form an antitumor phenotype with more antitumor immune cells and immunostimulatory-related cytokines.

We next sought to investigate the correlation between infiltrating immune cells and immune checkpoints TIGIT, CD48, PDCD1 (PD-1), LAG3, CD274, HAVCR2, LAIR1, and PDCD1LG2(PD-L2). The result elucidated that infiltrating immune cells were positively correlated with all above immune checkpoints (Figure 2(h), cor > 0.6). Notably, the correlation coefficients with CD48, HAVCR2, and PDCD1LG2 were more than 0.8, suggesting that EOC patients with high infiltrating immune cells may be more sensitive to ICBs therapy.

### 3.3. Dendritic Cells Shape the Immune Phenotype of EOC Microenvironment

**Figure 3:**
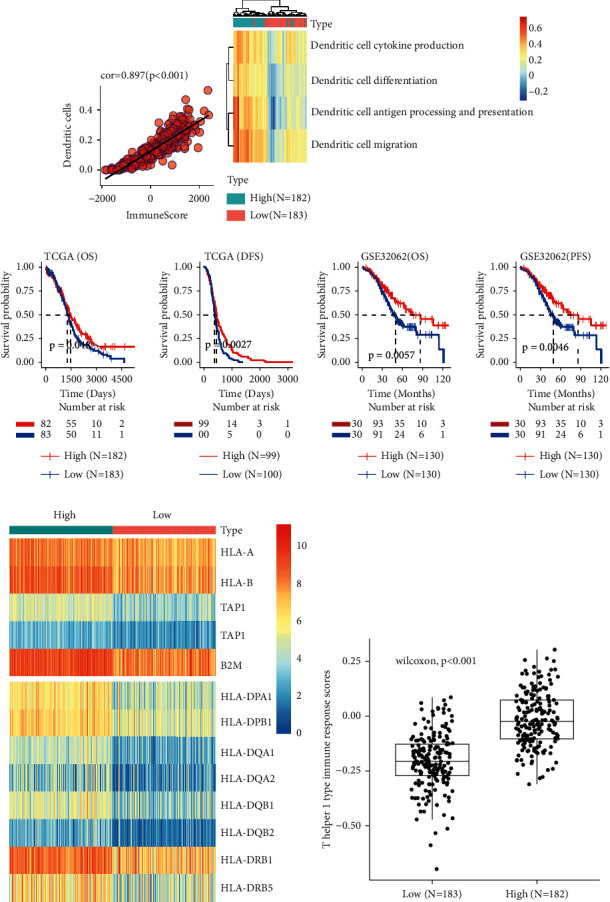
Dendritic cells shape the immune phenotype of EOC microenvironment. (a) Spearman's correlation analysis shows that the immune score is highly positively correlated with DCs infiltration (cor = 0.896, *p*=0.001). (b) Heatmap shows the enrichment of DC-related biological process between the high- and low-immune-score group. (c) K-M analyses show that EOC patients with high infiltrating DC had longer OS, DFS, and PFS. (d) Heatmap displaying the MHC-I/II molecules differentially expressed in high-immune-score group. (e) Box plot of ssGSEA for Th1-type immune response. OS: overall survival; DFS: disease-free survival; PFS: progression-free survival.

The correlation between infiltrating immune cells and different types of immune cell was assessed by Spearman's correlation analysis, and the results showed that the DCs were strongly positively correlated with immune score (Figure 3(a), cor = 0.896, *p* < 0.001). Meanwhile, the enrichment levels of DC-related biological processes were high in patients with high immune score (Figure 3(b)). It is worth mentioning that cytokines *CCL4*, *CXCL9*, and *CXCL10*, which were highly expressed in the high infiltrating immune cells group, can attract DCs (Figure 2(f)). In addition, K-M survival analysis indicated that the patients with high infiltrating DCs presented significantly better clinical survival (Figure 3(c)).

DCs are crucial participates in antigen presentation, activating MHC-I-restricted CTL responses and MHC-II-restricted CD4 + Th1 responses, both ultimately contributes to antitumor response. As shown in heatmap, almost all the MHC-I molecules (HLA-A, HLA-B, TAP1, TAP2, and B2M) and MHC-II molecules (HLA-DPA1, HLA-DPB1, HLA-DQA1, HLA-DQA2, HLA-DQ81, HLA-DQB2, HLA-DRB1, and HLA-DRB5) were overexpressed in high-immune-score group (Figure 3(d)). Furthermore, high infiltrating group ranked the higher score in T helper 1 type immune response (Figure 3(e)). These suggest that DCs may play a dominant role to promoting the infiltration of immune cells into tumor microenvironment and forming an antitumor immune phenotype.

### 3.4. Construction and Validation of Dendritic Cell-Related Risk Model

**Figure 4:**
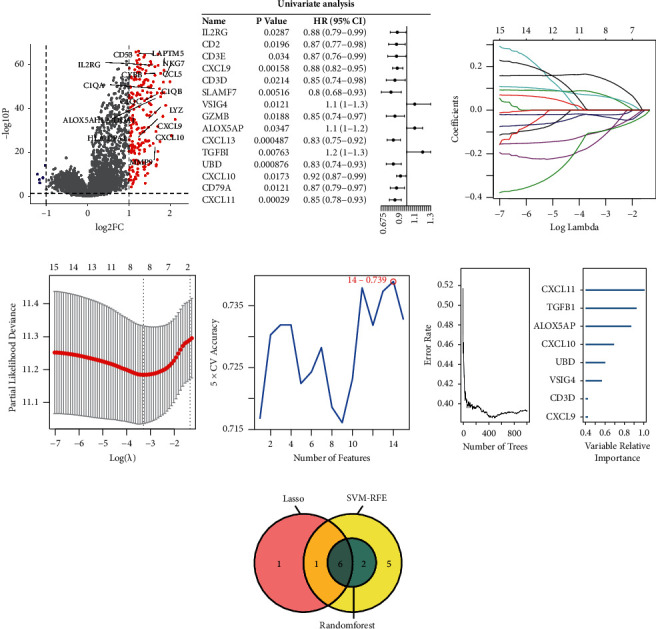
Identification of dendritic cells-related prognostic biomarkers (a) The volcano plot of all genes, the red dots represent 119 upregulated DEGs with |log FC| > 1 and *p* < 0.05, and the blue dot represents one downregulated DEG. (b) 15 candidate genes in the univariate Cox regression analysis. (c) Lasso coefficient profiles of 15 candidate genes. (d) Partial likelihood deviance of Lasso regression coefficients, Lasso regression with tenfold cross-validation obtains eight prognostic genes using minimum *λ* value. (e) Identification of optimal prognostic genes for EOC through SVM. (f) Left panel shows the relationship between the number of trees and error rate. The right panel shows that the random forest algorithm was used to further select DC-related prognostic biomarkers. (g) Venn diagram shows the overlapped DC-related prognostic biomarkers between Lasso regression, SVM and random forest. SVM: support vector machine.

As shown in volcano plot, a total of 120 genes (including 119 upregulated and 1 downregulated genes) were found to be differently expressed in high infiltrating DC group (Figure 4(a), |log FC| > 1, *p* < 0.05). Then, 15 out of 120 DEGs were screened as the OS-related DEGs via univariate Cox analysis (Figure 4(b)). Afterward, the machine-learning Lasso regression, SVM, and random forest were employed to identify the important factors (Figure 4(c)–4(f)). Collectively, six DC-related prognostic genes *CXCL9*, *VSIG4*, *ALOX5AP*, *TGFBI*, *UBD,* and *CXCL11* were identified to establish a risk model for EOC (Figure 4(g)). Three of them (*CXCL9*, *UBD*, and *CXCL11*) were protective genes with hazard ratio (HR) < 1, and others (*VSIG4*, *ALOX5AP*, and *TGFBI*) were risky genes (Figure 4(b)).

**Figure 5:**
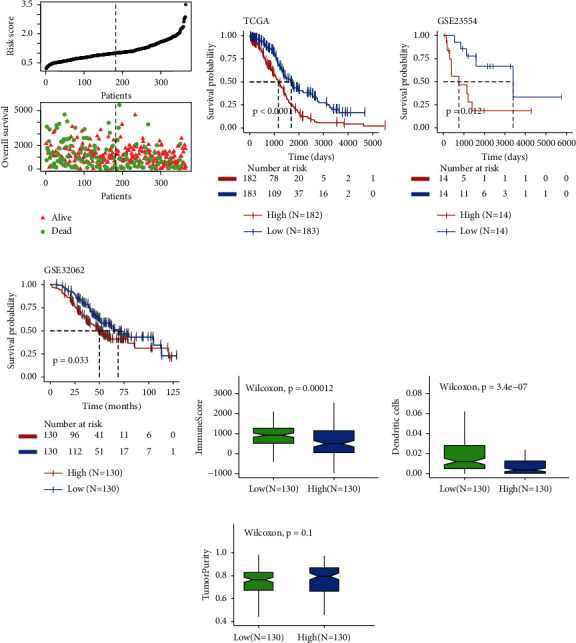
Construction and validation of dendritic cell-related risk model. (a) The distribution of risk scores in the TCGA cohort. (b–d) K-M curves of the OS according to risk scores in (b) the training cohort and validated cohorts (c) GSE23554, (d) GSE32062. (e–g) The distribution of the (e) immune score, (f) infiltration level of DCs, and (g) tumor purity between high- and low-risk score group.

The DC-related risk model had been applied to TCGA cohort, which classified EOC patients into high- and low-risk groups based on median risk score (Figure 5(a)). Different OS was noticed between two subgroups (Figure 5(b), High_median OS_ vs. Low_median OS_: 1162 vs. 1680 days, *p* < 0.001). Moreover, the additional cohort GSE23554 (Figure 5(b), High_median OS_ vs. Low_median OS_: 760 vs. 3399 days, *p*=0.012) and GSE32062 (Figure 5(c), High_median OS_ vs. Low_median OS_: 50 vs. 69 months, *p*=0.033). We also confirmed that the risk score was significantly negatively correlated with infiltrating immune cells and DCs but not related to tumor purity (Figure 5(d)–5(f)).

### 3.5. Nomogram

**Figure 6:**
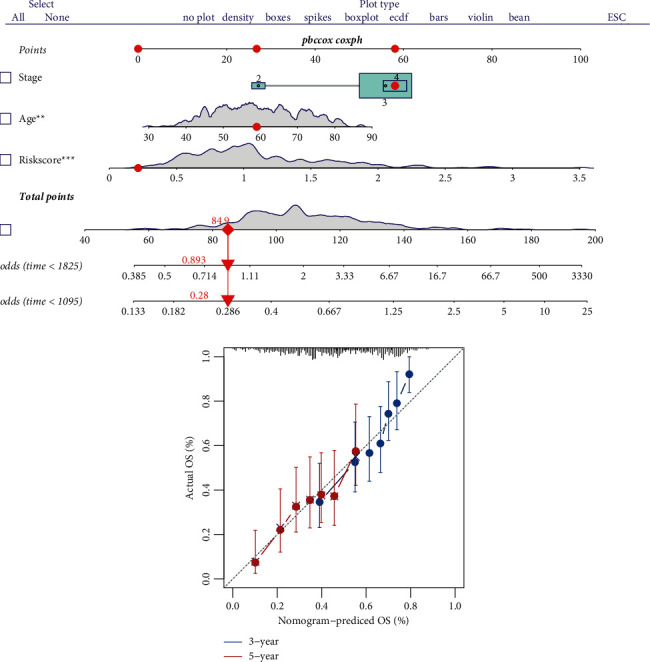
Nomogram. (a) Nomogram for predicting 3- and 5-year OS for EOC patients in TCGA dataset based on DC-related risk score and clinicopathological parameters (FIGO stage and age). (b) Calibration curves of nomogram in terms of agreement between predicted and actual 3- and 5-year outcomes in the TCGA cohort. The dashed line of 45° represents perfect prediction, and the actual performances of our nomogram are shown by green, red, and blue lines.

To analyze the relationship between the DC-related risk model and clinical parameters, the nomogram was developed based on clinical parameters (FIGO stage and Age) integrated with the risk score. The result illustrated that the risk score we constructed was an independent predictor in EOC (Figure 6(a)). Calibration curve showed that the nomogram has a good reliability in predicting 3- and 5-year survival (Figure 6(b)).

## 4. Discussion

ICB therapy has been a hot topic in tumor immunotherapy and is expected to improve the outcomes of EOC patients with relapse or chemoresistance [29–31]. However, ICB's clinical application in EOC is limited seriously because the TME is complex and has not been well investigated in EOC. The work flow of this work is shown in Supplementary Figure 3; we systematically analyzed the potential role and underlying mechanisms of infiltrating lymphocytes within EOC and dug the effective predictors for EOC prognosis and ICB's therapy.

In this study, we found that the immune cells were highly infiltrated into TME in carcinogenesis and predict a better clinical outcome in EOC, which are consistent with the study by Hao et al. [32], that the immune score can be used as a powerful predictive tool for both prognosis and chemotherapeutic sensitivity of EOC.

As we all know, TME phenotype was determined by a complex regulatory network involving antitumor immune responses, tumor escape, and cellular and molecular characteristics. In the current work, an antitumor phenotype with more antitumor immune cells and immunostimulatory-related cytokines was observed in high infiltrating lymphocytes group. In addition, consistent with Nishino et al. [4, 33], we also observed a strong positive correlation between infiltrating immune cells and immune checkpoints, such as CD48, HAVCR2 and PDCD1LG2, which is an important intrinsic immune surveillance escape mechanism; the PD-1, CTLA-4, BTLA, HAVCR2, and Lag3 have been proved to be the potent immune checkpoints to mediate tumor immunosuppression [34, 35]. Among which, predominately centered on PD-L1 that overexpressed in tumor cells, leading to inhibition of CTLs' proliferation [36]. Single-agent PD-1 blockade and PD-1/PD-L1 dual-regimen therapy both exhibit longer PFS in persistent and recurrent EOC [37]. HAVCR2 is significantly overexpressed in CD4^＋^and CD8^＋^T cells and suppress the antitumor immune responses in primary ovarian cancer, the blockade of HAVCR2 leads to sustained antitumor reactions [38]. This study suggests that EOC patients with high infiltrating immune cells may be able to achieve better efficacy in ICB's therapy.

In addition, we observed that DCs might highly express MHC-I and MHC-II molecules, then initiating MHC-I-restricted CTLs responses and MHC-II-restricted CD4^+^ Th1 responses, which enables T-cell immune response [39]. All in all, DCs may play a dominant role in promoting the infiltration of immune cells into TME and forming an antitumor immune phenotype. The MHC-I/II CTLs and Th1 immune responds were activated by DCs, both can further enhance the antitumor immunity to kill the tumor cells.

Immunological epigenetic alterations have already become innovative and precise cancer biomarkers in urologic, brain, lung, breast, and colorectal cancers [40–42]. However, in EOC with highly heterogeneous, the traditional tumor, node, and metastasis (TNM) classification are not efficient for prognostic assessment [43]. In this study, six DC-related prognostic biomarkers (*CXCL9*, *VSIG4*, *ALOX5AP*, *TGFBI*, *UBD*, and *CXCL11*) were identified to construct risk model, which could accurately stratify the EOC patients into two subtypes with different survival outcomes, providing an informative prognostic assessment for EOC patients. We hypothesize these molecules may play important regulatory role in EOC. *CXCL9* and *CXCL11* are known for their tumor suppressive properties and are expressed on the DCs; *CXCL9* and *CXCL11* have been described to enhance antitumor immunity by activating Th1 [46]. *UBD* (ubiquitin D) also known as FAT10, is induced by mature DCs and contributes to antigen presentation [47]. As for risky genes, VSIG4 (V‐set and Ig containing 4), a transmembrane receptor, was expressed specifically in DCs and macrophages, which inhibited the T cells' proliferation and immune response [48, 49]. The activation of *ALOX5AP* (Arachidonate 5-lipoxygenase activating protein) correlates with the HER2 (human epidermal growth factor receptor 2) and promotes the growth and migration of breast cancer [50]. *TGFBI* (transforming growth factor beta induced) has been reported to be an oncogene in a variety of cancers including prostate, ovarian, and breast cancer [51–53]. Fico and Santamaria‐Martínez found that *TGFBI* induced breast cancer metastasis by regulating the TME and hypoxia [51]. The combination of interstitial *TGFBI* and intratumoral CD8^+^ T cells can be used as a predictor for PD-1/L1 blockade nivolumab [54].

## 5. Conclusion

In summary, our results indicate that the heavily infiltrated immune cells were positively related to a better outcome and antitumor phenotype in EOC. ICBs therapy should be considered for EOC patients with high infiltrating immune cells. In addition, we provided the mechanistic insights in this study, showing that the DCs may play an important role in the initiation of antitumor immune. Finally, we verified that the established DC-related risk model is able to accurately stratify patients into subgroups with different survival outcomes. Nevertheless, further validation studies of these molecular mechanisms are still warranted in the future.

## Data Availability

Previously reported sequencing data were used to support this study and are available at TCGA (https://www.cancer.gov/) and GEO (http://www.ncbi.nlm.nih.gov/geo). These prior studies and datasets are cited at relevant places within the text as references [14–16].

## Supplementary Materials

Supplementary Materials Supplementary Figure 1: the box plot shows that the BRCA1/2 mutation has no significant correlation with immune score (t-test, *p*=0.271). Supplementary Figure 2: time-dependent ROC curves in (left) GSE32062 and (right) GSE63885 indicating high accuracy of immune score in OS prediction. ROC: receiver operating characteristic; OS: overall survival. Supplementary Figure 3: the work flow of this study. Supplementary Table 1: gene sets for gene set variation analysis.
