# Supplementary material for: Landscape of Immune Microenvironment in Epithelial Ovarian Cancer and Establishing Risk Model by Machine Learning
Source: J Oncol. 2021 Aug 26;2021:5523749. doi: 10.1155/2021/5523749 (PMC8416376; doi:10.1155/2021/5523749)
Supplement: Supplementary Materials — Supplementary Figure 1: the box plot shows that the BRCA1/2 mutation has no significant correlation with immune score (t-test, p=0.271). Supplementary Figure 2: time-dependent ROC curves in (left) GSE32062 and (right) GSE63885 indicating high accuracy of immune score in OS prediction. ROC: receiver operating characteristic; OS: overall survival. Supplementary Figure 3: the work flow of this study. Supplementary Table 1: gene sets for gene set variation analysis. [file 5523749.f1.zip › 5523749.f1/Supplementary table 1.docx]

**Supplementary table 1. Gene sets for gene set variation analysis.**

| GOBP ACTIVATION OF IMMUNE RESPONSE | *A2M, ABI1, ABL1, ACTB, ACTG1, ACTR2, ACTR3, ADA, AIM2, APCS, APPL1, APPL2, ARPC1A, ARPC1B, ARPC2, ARPC3, ARPC4, ARPC5, BAG6, BAIAP2, BAX, BCAR1, BCL10, BCL2, BLK, BLNK, BMX, BRK1, BTK, BTN1A1, BTN2A1, BTN2A2, BTN2A3P, BTN3A1, BTN3A2, BTN3A3, BTNL10, BTNL2, BTNL3, BTNL8, BTNL9, BTRC, C1QA, C1QB, C1QBP, C1QC, C1R, C1RL, C1S, C2, C3, C3AR1, C4A, C4B, C4BPA, C4BPB, C5, C5AR1, C5AR2, C6, C7, C8A, C8B, C8G, C9, CACNB3, CARD11, CARD9, CBFB, CBLB, CCR7, CD160, CD19, CD209, CD22, CD226, CD247, CD276, CD28, CD300A, CD38, CD3D, CD3E, CD3G, CD4, CD46, CD47, CD55, CD59, CD5L, CD79A, CD79B, CD81, CDC42, CEACAM1, CFB, CFD, CFH, CFHR1, CFHR2, CFHR4, CFHR5, CFI, CFP, CGAS, CHUK, CLEC10A, CLEC4A, CLEC4C, CLEC4D, CLEC4E, CLEC6A, CLU, CMKLR1, COLEC10, COLEC11, CPB2, CPN1, CPN2, CR1, CR1L, CR2, CREBBP, CRK, CRKL, CRP, CSK, CTLA4, CUL1, CYFIP1, CYFIP2, DENND1B, DGKZ, DOCK1, DUSP3, EIF2B1, EIF2B2, EIF2B3, EIF2B4, EIF2B5, ELF1, ELF2, ELMO1, ELMO2, EP300, ERMAP, EZR, F2, FBXW11, FCER1G, FCGR1A, FCGR2A, FCGR2B, FCGR3A, FCN1, FCN2, FCN3, FCRL3, FFAR2, FGR, FOXP1, FOXP3, FPR1, FPR2, FPR3, FYB1, FYB2, FYN, GATA3, GBP1, GCSAM, GCSAML, GPLD1, GPR32, GPR32P1, GPR33, GPS2, GRAP2, GRB2, HCK, HEXIM1, HHLA2, HLA-A, HLA-DPA1, HLA-DPB1, HLA-DQA1, HLA-DQA2, HLA-DQB1, HLA-DQB2, HLA-DRA, HLA-DRB1, HLA-DRB3, HLA-DRB4, HLA-DRB5, HMGB1, HMSD, HRAS, HSP90AA1, HSP90AB1, ICAM2, ICAM3, ICOSLG, IFI16, IGHA1, IGHA2, IGHD, IGHE, IGHG1, IGHG2, IGHG3, IGHG4, IGHM, IGHV1-18, IGHV1-24, IGHV1-3, IGHV1-45, IGHV1-58, IGHV1-69, IGHV1-69-2, IGHV1-69D, IGHV1OR15-1, IGHV2-26, IGHV2-5, IGHV2-70, IGHV2-70D, IGHV3-11, IGHV3-13, IGHV3-15, IGHV3-16, IGHV3-20, IGHV3-21, IGHV3-23, IGHV3-30, IGHV3-33, IGHV3-35, IGHV3-38, IGHV3-43, IGHV3-48, IGHV3-49, IGHV3-53, IGHV3-64, IGHV3-64D, IGHV3-66, IGHV3-7, IGHV3-72, IGHV3-73, IGHV3-74, IGHV4-28, IGHV4-31, IGHV4-34, IGHV4-39, IGHV4-4, IGHV4-59, IGHV4-61, IGHV5-10-1, IGHV5-51, IGHV6-1, IGHV7-4-1, IGHV7-81, IGKC, IGKV1-12, IGKV1-16, IGKV1-17, IGKV1-39, IGKV1-5, IGKV1D-12, IGKV1D-33, IGKV1D-39, IGKV2-28, IGKV2-29, IGKV2-30, IGKV2-40, IGKV2D-28, IGKV2D-30, IGKV3-15, IGKV3-20, IGKV3D-11, IGKV3D-20, IGKV4-1, IGKV5-2, IGLC1, IGLC2, IGLC3, IGLC6, IGLC7, IGLL1, IGLL5, IGLV1-40, IGLV1-44, IGLV1-47, IGLV1-51, IGLV2-11, IGLV2-14, IGLV2-23, IGLV2-8, IGLV3-1, IGLV3-19, IGLV3-21, IGLV3-25, IGLV3-27, IGLV6-57, IGLV7-43, IKBKB, IKBKG, IL1B, INPP5D, ITK, KCNN4, KHDRBS1, KIR2DS2, KLHL6, KLRC2, KLRD1, KRAS, KRT1, LAPTM5, LAT, LAT2, LAX1, LCK, LCP2, LGALS3, LILRA2, LILRB4, LIME1, LIMK1, LPXN, LYN, MALT1, MAP3K7, MAPK1, MAPK3, MASP1, MASP2, MATR3, MAVS, MBL2, MEF2C, MFAP4, MICB, MIR18A, MIR19A, MIR34A, MIR520B, MIR520E, MNDA, MOG, MS4A1, MUC1, MUC12, MUC13, MUC15, MUC16, MUC17, MUC19, MUC2, MUC20, MUC21, MUC3A, MUC4, MUC5AC, MUC5B, MUC6, MUC7, MUCL1, MYH2, MYO10, MYO1C, MYO1G, NCK1, NCKAP1, NCKAP1L, NCKIPSD, NCR3, NECTIN2, NFAM1, NFATC2, NFKB1, NFKBID, NFKBIZ, NLRC4, NOD2, NONO, NR4A3, NRAS, PAG1, PAK1, PAK2, PAK3, PAWR, PAX5, PDE4B, PDE4D, PDPK1, PHB, PHPT1, PIK3CA, PIK3CB, PIK3CD, PIK3R1, PIK3R2, PLA2G6, PLCG1, PLCG2, PLCL2, PLD2, PLEKHA1, PLPP4, PLSCR1, PQBP1, PRAM1, PRKACA, PRKACB, PRKACG, PRKCB, PRKCD, PRKCE, PRKCH, PRKCQ, PRKD2, PRKDC, PRNP, PROS1, PSEN1, PSMA1, PSMA2, PSMA3, PSMA4, PSMA5, PSMA6, PSMA7, PSMA8, PSMB1, PSMB10, PSMB11, PSMB2, PSMB3, PSMB4, PSMB5, PSMB6, PSMB7, PSMB8, PSMB9, PSMC1, PSMC2, PSMC3, PSMC4, PSMC5, PSMC6, PSMD1, PSMD10, PSMD11, PSMD12, PSMD13, PSMD14, PSMD2, PSMD3, PSMD4, PSMD5, PSMD6, PSMD7, PSMD8, PSMD9, PSME1, PSME2, PSME3, PSME4, PSMF1, PSPC1, PTK2, PTPN2, PTPN22, PTPN6, PTPRC, PTPRJ, PVRIG, PYCARD, PYHIN1, RAB29, RAC1, RAF1, RAP1A, RAPGEF1, RBCK1, RBM14, RC3H1, RC3H2, RELA, RELB, RFTN1, RGCC, RIPK2, RNF31, RPS3, RPS6KA5, RUNX1, SERPING1, SFPQ, SH2B2, SH2D1A, SIN3A, SKAP1, SKP1, SLA2, SLC39A10, SPG21, SPPL3, SRC, STAP1, STING1, STK11, STOML2, SUSD4, SYK, TAB1, TAB2, TAB3, TBK1, TEC, TESPA1, THEMIS, THEMIS2, THY1, TLR4, TNFRSF21, TOMM70, TRAC, TRAF6, TRAT1, TRAV19, TRAV29DV5, TRAV8-4, TRBC1, TRBC2, TRBV12-3, TRBV7-9, TRDC, TREX1, TRIM5, TXK, TYROBP, UBASH3A, UBE2N, VAV1, VAV2, VAV3, VSIG4, VTCN1, VTN, WAS, WASF2, WASL, WIPF1, WIPF2, WIPF3, WNK1, XRCC5, XRCC6, YES1, ZAP70, ZBP1, ZC3H12A, ZCCHC3* |
| --- | --- |
| GOBP ADAPTIVE IMMUNE RESPONSE | *ADA, ADAM17, ADCY7, ADGRE1, AGER, AIRE, ALCAM, ALOX15, ANXA1, APCS, ARG1, ARG2, AZGP1, B2M, BACH2, BATF, BCL10, BCL3, BCL6, BMX, BTK, BTLA, BTN3A1, BTN3A2, BTN3A3, BTNL8, C17orf99, C1QA, C1QB, C1QBP, C1QC, C1R, C1RL, C1S, C2, C3, C4A, C4B, C4BPA, C4BPB, C5, C6, C7, C8A, C8B, C8G, C9, CAMK4, CCL19, CCR2, CCR6, CD160, CD19, CD1A, CD1B, CD1C, CD1D, CD1E, CD209, CD226, CD244, CD247, CD27, CD274, CD28, CD3D, CD3E, CD3G, CD4, CD40, CD40LG, CD46, CD48, CD55, CD6, CD7, CD70, CD74, CD79A, CD79B, CD80, CD81, CD84, CD86, CD8A, CD8B, CD8B2, CEACAM1, CFI, CLC, CLCF1, CLEC10A, CLEC4A, CLEC4C, CLEC4D, CLEC4G, CLEC4M, CLEC6A, CLU, CR1, CR2, CRACR2A, CRP, CSK, CTLA4, CTSC, CTSH, CTSL, CTSS, CXCL13, CYRIB, DBNL, DCLRE1C, DENND1B, DUSP10, EBI3, EIF2AK4, EMP2, ENTPD7, EOMES, ERAP1, ERAP2, ERCC1, EXO1, EXOSC3, EXOSC6, FADD, FBXO38, FCAMR, FCER1G, FCER2, FCGR1B, FCGR2B, FCRL4, FGA, FGB, FGL1, FOXJ1, FOXP3, FUT7, FYN, FZD5, GAPT, GATA3, GNL1, GPR183, GZMM, HAVCR2, HFE, HLA-A, HLA-B, HLA-C, HLA-DMA, HLA-DMB, HLA-DOA, HLA-DOB, HLA-DPA1, HLA-DPB1, HLA-DQA1, HLA-DQA2, HLA-DQB1, HLA-DQB2, HLA-DRA, HLA-DRB1, HLA-DRB3, HLA-DRB4, HLA-DRB5, HLA-E, HLA-F, HLA-G, HLA-H, HLX, HMCES, HMGB1, HMHB1, HPRT1, HPX, HRAS, HSPD1, ICAM1, ICOSLG, IFNA1, IFNA10, IFNA13, IFNA14, IFNA16, IFNA17, IFNA2, IFNA21, IFNA4, IFNA5, IFNA6, IFNA7, IFNA8, IFNB1, IFNE, IFNG, IFNK, IFNW1, IGHA1, IGHA2, IGHD, IGHD1-1, IGHE, IGHG1, IGHG2, IGHG3, IGHG4, IGHJ1, IGHM, IGHV1-18, IGHV1-24, IGHV1-3, IGHV1-45, IGHV1-58, IGHV1-69, IGHV1-69-2, IGHV1-69D, IGHV1OR15-1, IGHV2-26, IGHV2-5, IGHV2-70, IGHV2-70D, IGHV3-11, IGHV3-13, IGHV3-15, IGHV3-16, IGHV3-20, IGHV3-21, IGHV3-23, IGHV3-30, IGHV3-33, IGHV3-35, IGHV3-38, IGHV3-43, IGHV3-48, IGHV3-49, IGHV3-53, IGHV3-64, IGHV3-64D, IGHV3-66, IGHV3-7, IGHV3-72, IGHV3-73, IGHV3-74, IGHV4-28, IGHV4-31, IGHV4-34, IGHV4-39, IGHV4-4, IGHV4-59, IGHV4-61, IGHV5-10-1, IGHV5-51, IGHV6-1, IGHV7-4-1, IGHV7-81, IGHV8-51-1, IGKC, IGKJ1, IGKV1-12, IGKV1-13, IGKV1-16, IGKV1-17, IGKV1-27, IGKV1-37, IGKV1-39, IGKV1-5, IGKV1-6, IGKV1-8, IGKV1-9, IGKV1D-12, IGKV1D-13, IGKV1D-17, IGKV1D-33, IGKV1D-37, IGKV1D-39, IGKV1D-42, IGKV1D-43, IGKV1D-8, IGKV2-24, IGKV2-28, IGKV2-29, IGKV2-30, IGKV2-40, IGKV2D-24, IGKV2D-26, IGKV2D-28, IGKV2D-29, IGKV2D-30, IGKV3-15, IGKV3-20, IGKV3-7, IGKV3D-11, IGKV3D-15, IGKV3D-20, IGKV3D-7, IGKV4-1, IGKV5-2, IGKV6-21, IGKV6D-21, IGKV6D-41, IGLC1, IGLC2, IGLC3, IGLC6, IGLC7, IGLJ1, IGLL1, IGLL5, IGLV1-36, IGLV1-40, IGLV1-44, IGLV1-47, IGLV1-50, IGLV1-51, IGLV10-54, IGLV11-55, IGLV2-11, IGLV2-14, IGLV2-18, IGLV2-23, IGLV2-33, IGLV2-8, IGLV3-1, IGLV3-10, IGLV3-12, IGLV3-16, IGLV3-19, IGLV3-21, IGLV3-22, IGLV3-25, IGLV3-27, IGLV3-32, IGLV3-9, IGLV4-3, IGLV4-60, IGLV4-69, IGLV5-37, IGLV5-45, IGLV5-48, IGLV5-52, IGLV6-57, IGLV7-43, IGLV7-46, IGLV8-61, IGLV9-49, IL10, IL12A, IL12B, IL12RB1, IL13RA2, IL17A, IL17F, IL17RA, IL18, IL18BP, IL18R1, IL1B, IL1R1, IL1RL1, IL2, IL20RB, IL23A, IL23R, IL27, IL27RA, IL33, IL4, IL4I1, IL4R, IL6, IL6R, IL6ST, IL7R, INPP5D, IRF1, IRF4, IRF7, ITK, JAG1, JAK2, JAK3, JAM3, JCHAIN, KLHL6, KLRC1, KLRC4-KLRK1, KLRD1, KLRK1, KMT5B, KMT5C, LAG3, LAIR1, LAMP3, LAT, LAT2, LAX1, LEF1, LIG4, LILRA1, LILRA3, LILRA6, LILRB1, LILRB2, LILRB3, LILRB4, LILRB5, LIME1, LOXL3, LTA, LY9, LYN, MAD2L2, MALT1, MAP3K7, MARCHF8, MASP2, MBL2, MCOLN1, MCOLN2, MEF2C, MICA, MICB, MIR21, MLH1, MR1, MSH2, MSH6, MTOR, MYD88, MYO1G, NBN, NDFIP1, NECTIN2, NFKB2, NFKBID, NFKBIZ, NLRP10, NLRP3, NOD2, NSD2, ORAI1, OTUB1, OTUD7B, PAG1, PARP3, PAXIP1, PDCD1, PDCD1LG2, PHB, PIK3CD, PIK3CG, PKN1, PLA2G4A, PPP3CB, PRDM1, PRF1, PRKCB, PRKCD, PRKCQ, PRKCZ, PRKD2, PRR7, PTK2B, PTPN6, PTPRC, PVR, PYCARD, RAB27A, RAG1, RC3H1, RC3H2, RELB, RFTN1, RIF1, RIPK2, RIPK3, RNF125, RNF168, RNF19B, RNF8, RORA, RORC, RSAD2, SAMSN1, SASH3, SCART1, SEMA4A, SERPING1, SH2D1A, SH2D1B, SHLD1, SHLD2, SHLD3, SIGLEC10, SIRT1, SIT1, SKAP1, SLA2, SLAMF1, SLAMF6, SLAMF7, SLC11A1, SLC15A4, SLC22A13, SMAD7, SOCS5, SPN, STAT3, STX7, SUPT6H, SUSD4, SWAP70, SYK, TAP1, TAP2, TARM1, TBX21, TCIRG1, TEC, TFE3, TFEB, TFRC, TGFB1, THEMIS, THOC1, TLR4, TLR8, TMEM98, TNF, TNFAIP3, TNFRSF11A, TNFRSF13B, TNFRSF13C, TNFRSF14, TNFRSF17, TNFRSF1B, TNFRSF21, TNFSF13, TNFSF13B, TNFSF18, TNFSF4, TP53BP1, TRAC, TRAF2, TRAF6, TRAJ3, TRAJ31, TRAJ42, TRAT1, TRAV1-1, TRAV1-2, TRAV10, TRAV12-1, TRAV12-2, TRAV12-3, TRAV13-1, TRAV13-2, TRAV14DV4, TRAV16, TRAV17, TRAV18, TRAV19, TRAV2, TRAV20, TRAV21, TRAV22, TRAV23DV6, TRAV24, TRAV25, TRAV26-1, TRAV26-2, TRAV27, TRAV29DV5, TRAV3, TRAV30, TRAV34, TRAV35, TRAV36DV7, TRAV38-1, TRAV38-2DV8, TRAV39, TRAV4, TRAV40, TRAV41, TRAV5, TRAV6, TRAV7, TRAV8-1, TRAV8-2, TRAV8-3, TRAV8-4, TRAV8-6, TRAV9-1, TRAV9-2, TRBC1, TRBC2, TRBD1, TRBJ1-1, TRBJ1-2, TRBJ1-3, TRBJ1-4, TRBJ1-5, TRBJ1-6, TRBJ2-1, TRBJ2-2, TRBJ2-3, TRBJ2-4, TRBJ2-5, TRBJ2-6, TRBJ2-7, TRBV10-1, TRBV10-2, TRBV10-3, TRBV11-1, TRBV11-2, TRBV11-3, TRBV12-3, TRBV12-4, TRBV12-5, TRBV13, TRBV14, TRBV16, TRBV17, TRBV18, TRBV19, TRBV2, TRBV20-1, TRBV23-1, TRBV24-1, TRBV25-1, TRBV27, TRBV28, TRBV29-1, TRBV3-1, TRBV30, TRBV4-1, TRBV4-2, TRBV5-1, TRBV5-3, TRBV5-4, TRBV5-5, TRBV5-6, TRBV5-7, TRBV6-1, TRBV6-4, TRBV6-5, TRBV6-6, TRBV6-7, TRBV6-8, TRBV7-1, TRBV7-2, TRBV7-3, TRBV7-4, TRBV7-6, TRBV7-7, TRBV7-9, TRBV9, TRDC, TRDD1, TRDJ1, TRDV1, TRDV2, TRDV3, TREX1, TRGC2, TRGJ2, TRGV1, TRGV10, TRGV11, TRGV2, TRGV3, TRGV4, TRGV5, TRGV8, TRGV9, TRIM27, TRPM4, TSC1, TXK, UNC13D, UNC93B1, UNG, VTCN1, WAS, XCL1, ZAP70, ZBTB1, ZBTB7B, ZC3H12A, ZNF683, ZP3* |
| GOBP ACTIVATION OF INNATE IMMUNE RESPONSE | *AIM2, BCL10, BTRC, CARD11, CARD9, CD209, CGAS, CHUK, CLEC10A, CLEC4A, CLEC4C, CLEC4D, CLEC4E, CLEC6A, CREBBP, CUL1, EP300, FBXW11, FCER1G, FCN1, FFAR2, FYN, HCK, HEXIM1, HMGB1, HRAS, HSP90AA1, ICAM2, ICAM3, IFI16, IKBKB, IKBKG, KIR2DS2, KLRC2, KLRD1, KRAS, LILRA2, LYN, MALT1, MAP3K7, MATR3, MAVS, MNDA, MUC1, MUC12, MUC13, MUC15, MUC16, MUC17, MUC19, MUC2, MUC20, MUC21, MUC3A, MUC4, MUC5AC, MUC5B, MUC6, MUC7, MUCL1, NFKB1, NLRC4, NONO, NRAS, PAK1, PAK2, PAK3, PDPK1, PLCG2, PQBP1, PRKACA, PRKACB, PRKACG, PRKCD, PRKDC, PSMA1, PSMA2, PSMA3, PSMA4, PSMA5, PSMA6, PSMA7, PSMA8, PSMB1, PSMB10, PSMB11, PSMB2, PSMB3, PSMB4, PSMB5, PSMB6, PSMB7, PSMB8, PSMB9, PSMC1, PSMC2, PSMC3, PSMC4, PSMC5, PSMC6, PSMD1, PSMD10, PSMD11, PSMD12, PSMD13, PSMD14, PSMD2, PSMD3, PSMD4, PSMD5, PSMD6, PSMD7, PSMD8, PSMD9, PSME1, PSME2, PSME3, PSME4, PSMF1, PSPC1, PYCARD, PYHIN1, RAF1, RBM14, RELA, RELB, RPS6KA5, SFPQ, SIN3A, SKP1, SRC, STING1, SYK, TAB1, TAB2, TAB3, TBK1, TLR4, TOMM70, TRAF6, TRIM5, TYROBP, XRCC5, XRCC6, ZBP1, ZCCHC3* |
| GOBP DENDRITIC CELL CYTOKINE PRODUCTION | *BST2, CLEC7A, DDX21, DDX58, DHX36, JAK3, KIT, MAVS, NOD2, SCIMP, TICAM1, TLR3, TLR4* |
| GOBP DENDRITIC CELL DIFFERENTIATION | *AGER, AXL, AZI2, BATF, BATF2, BATF3, BLK, CAMK4, CCL19, CCR7, CEBPB, CSF2, DCSTAMP, DHRS2, F2RL1, FCGR2B, FLT3, GAS6, GATA1, HLA-B, HLA-G, HMGB1, IL4, IRF4, IRF8, ITGB6, ITGB8, LGALS9, LILRB1, LILRB2, LTBR, LYN, MIR223, NOTCH2, PRTN3, PSEN1, RBPJ, RELB, SPI1, TGFBR2, TMEM176A, TMEM176B, TRAF6, TREM2, TRPM2, UBD, ZBTB46* |
| GOBP DENDRITIC CELL ANTIGEN PROCESSING AND PRESENTATION | *CCL19, CCL21, CCR7, CD68, CD74, CLEC4A, FCGR2B, FGL2, HLA-DRA, HLA-DRB1, HLA-DRB3, NOD1, NOD2, SLC11A1, THBS1* |
| GOBP DENDRITIC CELL MIGRATION | *ALOX5, ANO6, C1QBP, CALR, CCL19, CCL21, CCL5, CCR1, CCR2, CCR5, CCR6, CCR7, CDC42, CXCR1, CXCR2, CXCR4, DOCK8, EPS8, EXT1, GAS6, GPR183, HMGB1, IL12A, LGALS9, PIK3CG, SLAMF8, TNFSF18, TRPM2, TRPM4* |
| GO POSITIVE REGULATION OF T HELPER 1 TYPE IMMUNE RESPONSE | *ANXA1, CCL19, CCR2, CD80, HLX, IL12B, IL12RB1, IL18, IL18R1, IL1B, IL1R1, IL23A, IL23R, IL27RA, NLRP10, PLA2G4A, RIPK2, SLC11A1, SOCS5, XCL1* |
