# Supplementary figures and images for: Landscape of Immune Microenvironment in Epithelial Ovarian Cancer and Establishing Risk Model by Machine Learning
Source: J Oncol. 2021 Aug 26;2021:5523749. doi: 10.1155/2021/5523749 (PMC8416376; doi:10.1155/2021/5523749)

# Supplementary figure 1

## BRCA1/2(ns)

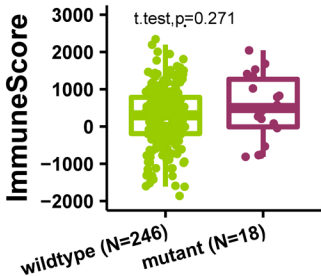

Supplement: Supplementary Materials — Supplementary Figure 1: the box plot shows that the BRCA1/2 mutation has no significant correlation with immune score (t-test, p=0.271). Supplementary Figure 2: time-dependent ROC curves in (left) GSE32062 and (right) GSE63885 indicating high accuracy of immune score in OS prediction. ROC: receiver operating characteristic; OS: overall survival. Supplementary Figure 3: the work flow of this study. Supplementary Table 1: gene sets for gene set variation analysis. [file 5523749.f1.zip › 5523749.f1/Supplementary figure 1 (2).pdf]

## Supplementary figure 2

### GSE 32062

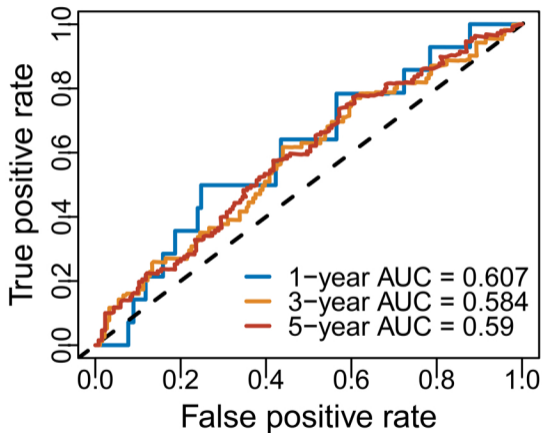

### GSE63885

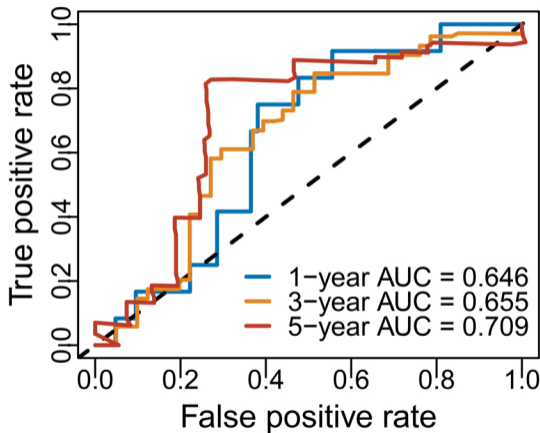

Supplement: Supplementary Materials — Supplementary Figure 1: the box plot shows that the BRCA1/2 mutation has no significant correlation with immune score (t-test, p=0.271). Supplementary Figure 2: time-dependent ROC curves in (left) GSE32062 and (right) GSE63885 indicating high accuracy of immune score in OS prediction. ROC: receiver operating characteristic; OS: overall survival. Supplementary Figure 3: the work flow of this study. Supplementary Table 1: gene sets for gene set variation analysis. [file 5523749.f1.zip › 5523749.f1/Supplementary figure 2 (2).pdf]

Supplementary figure 3

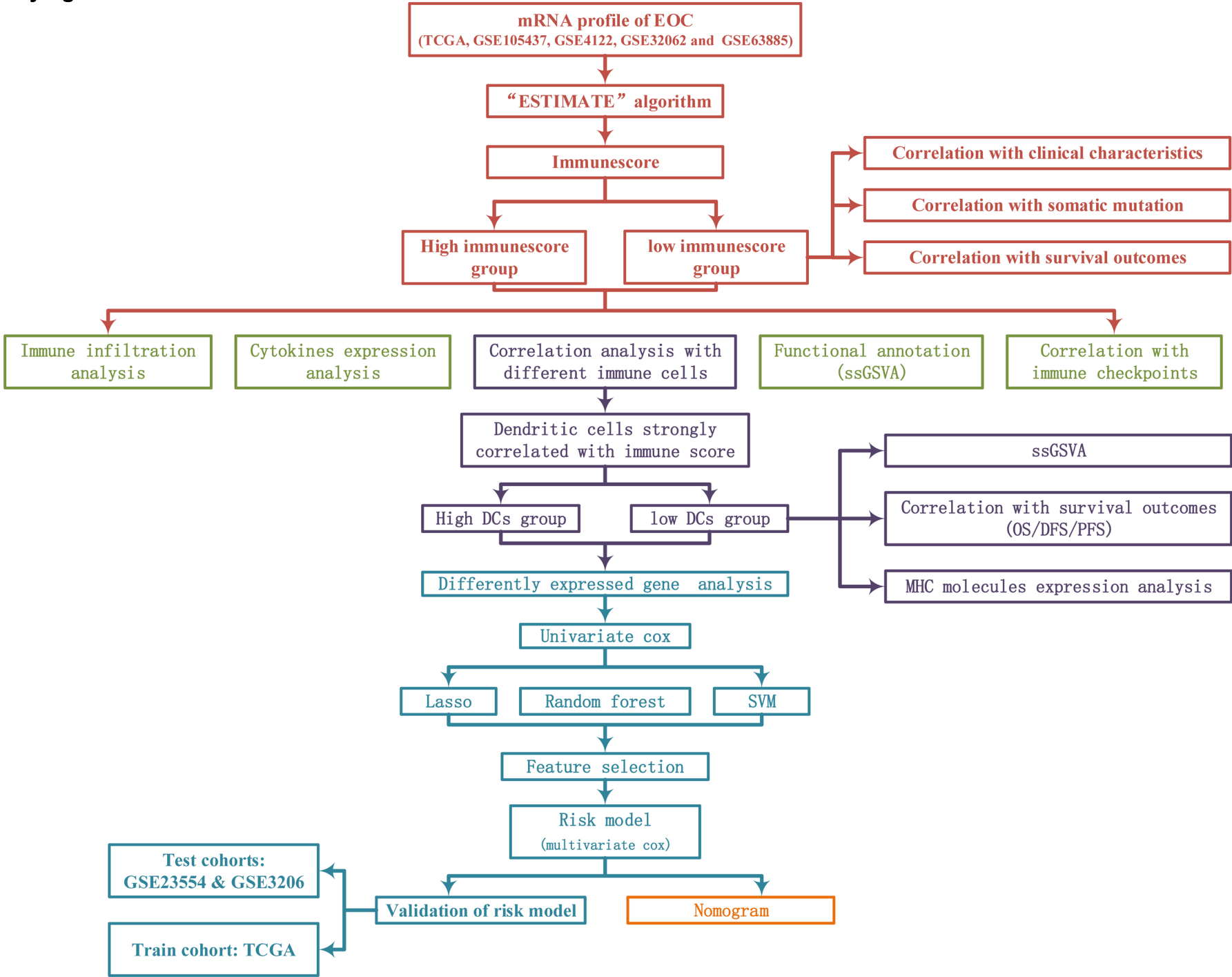

Supplement: Supplementary Materials — Supplementary Figure 1: the box plot shows that the BRCA1/2 mutation has no significant correlation with immune score (t-test, p=0.271). Supplementary Figure 2: time-dependent ROC curves in (left) GSE32062 and (right) GSE63885 indicating high accuracy of immune score in OS prediction. ROC: receiver operating characteristic; OS: overall survival. Supplementary Figure 3: the work flow of this study. Supplementary Table 1: gene sets for gene set variation analysis. [file 5523749.f1.zip › 5523749.f1/Supplementary figure 3 (2).pdf]
